# Assessment of Receiver Signal Strength Sensing for Location Estimation Based on Fisher Information

**DOI:** 10.3390/s16101570

**Published:** 2016-09-24

**Authors:** John Nielsen, Christopher Nielsen

**Affiliations:** Department of Electrical and Computer Engineering, University of Calgary, Calgary, AB T2N-1N4, Canada; chris@appropolis.com

**Keywords:** indoor localization, Wi-Fi fingerprinting, wireless, localization techniques, FIM, RSS, received signal strength, radio map, fisher information, Cramer Rao bound (CRB)

## Abstract

Currently there is almost ubiquitous availability of wireless signaling for data communications within commercial building complexes resulting in receiver signal strength (RSS) observables that are typically sufficient for generating viable location estimates of mobile wireless devices. However, while RSS observables are generally plentiful, achieving an accurate estimation of location is difficult due to several factors affecting the electromagnetic coupling between the mobile antenna and the building access points that are not modeled and hence contribute to the overall estimation uncertainty. Such uncertainty is typically mitigated with a moderate redundancy of RSS sensor observations in combination with other constraints imposed on the mobile trajectory. In this paper, the Fisher Information (FI) of a set of RSS sensor observations in the context of variables related to the mobile location is developed. This provides a practical method of determining the potential location accuracy for the given set of wireless signals available. Furthermore, the information value of individual RSS measurements can be quantified and the RSS observables weighted accordingly in estimation combining algorithms. The practical utility of using FI in this context was demonstrated experimentally with an extensive set of RSS measurements recorded in an office complex. The resulting deviation of the mobile location estimation based on application of weighted likelihood processing to the experimental RSS data was shown to agree closely with the Cramer Rao bound determined from the FI analysis.

## 1. Introduction

Associated with the proliferation of smart phones, tablets and wireless sensors is a dense collection of wireless signal transmissions that are either sourced or received by a mobile wireless device (MWD). Coupled with each wireless signal is a measure of the receiver signal strength (RSS) supplied by the wireless receiver as a byproduct of the processing required to facilitate data communications. As the RSS observables are related and sensitive to the location of the MWD they provide exploitable information in this regard. A comprehensive summary of MWD location estimation techniques based on RSS observables is presented in [1,2] which is not the focus of this paper. Rather, the interest here is in quantifying the information contained in the set of RSS observables in the context of the MWD location based on evaluating the Fisher Information (FI).

Assessment of the FI related to a set of observables, can be transformed directly into the Cramer Rao Bound (CRB) which specifies the lower bound of the covariance of an unbiased estimator of a set of parameters [3,4]. FI and CRB analysis has been fundamental to the estimation discipline for decades and is clearly applicable to the present application which is relating the accuracy of an MWD location estimator to the set of RSS observables. However, while there are some publications that refer to the FI related to RSS observables [5,6], there are additional and fundamental insights that can be gleaned. The objective of this paper is primarily to provide a more unified approach for the analysis of the FI over a set of RSS observables in the MWD location estimation context and in that process illuminate insights that have, in the authors’ opinion, hitherto been obscured. Additionally, the objective is to present FI analysis as a practical tool for assessing the value of a set of RSS measurements. This is based on abstracting the FI from the set of RSS fingerprinting radio maps or empirical radio propagation models that are a prerequisite for MWD location estimation in a given building environment. From the FI, the optimal achievable performance of an MWD location estimation algorithm can be determined without specifying the algorithm itself. Based on this, it is possible to determine if the set of wireless signals available to the MWD is sufficient for a given specification of location accuracy or if further infrastructure augmentation is required such as adding an additional wireless access point (AP). This will be shown based on idealized analysis followed by an experimental assessment conducted on an extensive set of RSS measurements taken inside an office complex.

The generic MWD location estimation algorithm based on RSS observables utilizes some form of weighted combining of these observables. It is intuitive that the FI calculated for each RSS observable is useful in determining such a weighting scheme, such that RSS observables of higher FI are weighted more than RSS observables with lower FI. Typical deployed combining schemes are based instead on the RSS level directly such that stronger received signals are weighted more than weaker signals. Of interest is that this is essentially equivalent to weighting proportional to the quantity of FI as will be explained which is a novelty of the paper. Summarizing, the contribution of this paper is the intuition and insight gained from considering RSS observables from the perspective of FI which additionally results in a more unified approach to constructing and justifying combining algorithms as well as providing an upper bound on the achievable location estimation accuracy.

The outline for the remainder of the paper is as follows. In Section 2 further background into the MWD location estimation based on RSS observables is provided with an introduction to the FI analysis of the RSS observables. In Section 3, FI analysis is applied to a two dimensional location estimation based on RSS observables from multiple APs and with a simplified empirical path loss model. In Section 4 experimental RSS measurement results for 2.4 GHz and 5 GHz Wi-Fi of an office building are presented and analyzed with three objectives. The first is to quantify the various factors of uncertainty in the RSS measurements which is needed for the FI calculation. The second is to give a practical method of calculating the FI from the surveyed RSS throughout the office area. The third is to demonstrate the agreement between the CRB of the MWD location estimation and deviation of the maximum likelihood estimator (MLE) based on RSS observables. Section 5 concludes the paper with a summary of the findings and contributions of the paper.

## 2. Fisher Information Formulation

In a typical airport lobby, shopping mall, or university common area, there are literally hundreds of individual wireless signals, each representing the opportunity for an independent RSS observation. As stated, RSS observations are readily available in the receiver such that the MWD location estimate can be achieved with very little additional computation [7,8,9]. Unfortunately, achieving location accuracy of better than several meters is difficult as the RSS is subject to many factors [10,11,12,13]. The most significant are as follows: (1) path loss attenuation; (2) excess penetration losses; (3) signal shadowing [14]; (4) small scale multipath that causes variations of up to 20 dB over a short distance of half a wavelength of the carrier; (5) orientation and position on the MWD; (6) body absorption of the person carrying the MWD or other persons in close proximity [15]; (7) general radio interference that impairs the ability of the receiver to generate an accurate RSS reading [16,17,18]; and (8) variable output power of wireless routers which are sometimes configured to adapt to fluctuating data traffic. The information of the RSS observation and ultimately the accuracy of the MWD location estimate depends on if and how well these factors can be modeled. Basically, whatever aspect of RSS that can be deterministically modeled is useful as a signal quantity while factors that can only be represented statistically contribute to the uncertainty or “noise” component of the measurement. For example, if the radio shadowing of an object in a building is modeled then the effect of the shadowing becomes a useful signal feature of the RSS field that contributes to the FI of the RSS observation. However, if the shadowing effect of the building object is not modeled then it becomes a source of uncertainty that reduces the effective information provided by the RSS observation. If it is neither represented as a feature or an uncertainty, then it can result in significant unexpected bias error in the MWD location estimate. As another example, consider the small scale multipath which is not practical to model as it is highly sensitive to any minute change in the building propagation environment. As it is impractical to model, it remains as a source of uncertainty in the RSS observation and is represented statistically with a random variable as will be detailed in Section 4.

Features such as small scale multipath that are represented by a random variable typically decorrelate with changes in MWD antenna location, carrier frequency and so forth such that uncertainty can be mitigated with averaging over a sufficiently large set of RSS observations. In other words, a partial marginalization of the non-modeled random components of the RSS observations is used to enhance the FI of the observations. In a typical Wi-Fi AP transmission there are 2.4 GHz and 5 GHz bands that each extend over 80 MHz, which significantly exceed the coherence bandwidth of the indoor channel and provide an important source of sample diversity facilitating such marginalization. Sequential RSS measurements as the MWD is moved is another source of diversity. For instance, in typical Rayleigh fading due to small scale multipath, the RSS decorrelates over a distance on the order of half the carrier wavelength which is only several centimeters in the Wi-Fi bands. Hence a relatively large set of RSS observables from the same Wi-Fi channel is available over a modest trajectory length of the MWD of several meters [19,20].

Consider the RSS in a typical building environment which we will regard as a multi-dimensional scalar field referred to herein as the RSS field. For the sake of simplicity, we initially ignore the dependence of the RSS field on height from the floor surface and orientation of the MWD antenna. Consequently, the RSS field is two dimensional referring to the two horizontal orthogonal spatial dimensions which when sampled through some prerequisite radio surveying becomes the radio map (RM). There are two varieties of the RM that will be considered, the sampled radio map (SRM) and the parametric radio map (PRM). There is one SRM or PRM for every AP generated and stored as an outcome of initial surveying or crowd sourcing. The SRM is essentially a spatially smoothed and interpolated version of the RM based directly on the surveyed RSS sampling. In the PRM, the main spatial features of the RM are represented by a parameterized empirical relation wherein the parameters are evaluated based on curve fitting methods applied to the surveyed RSS data. The simplest PRM would be a free space path loss model in which the RSS varies inversely as the square of the range between the AP and the MWD. More parameters can be included for a refined model, however, shadowing and structural related excess penetration losses are difficult to model empirically at which point the SRM becomes a better representation.

RMs whether of the PRM or SRM variety depend on the propagation environment remaining static [21,22,23]. Small changes such as a minor movement of an AP generally requires a new radio map calibration. In addition to leaving out the height and MWD antenna orientation from the RM, the other previously listed factors are of significance. For instance the issue of the AP transmitter power changing has been addressed based on relegating the transmitter power to be a nuisance parameter that is jointly estimated along with the MWD location. As for the location of the MWD on the person, many studies have considered using IMU sensors to provide gesture signatures that coupled with classifier algorithms can be used for determining if the MWD is in the shirt or pant pocket or carried in a dangling handbag [24,25,26,27,28]. Based on this additional information, probabilistic estimation of nuisance parameters can be used to provide temporary adjustments to the RM content before proceeding with the location estimation of the MWD.

The dimensional space of the RM can in principle be expanded to encompass all of the modeled factors affecting the RSS. The MLE type location estimation would then be extended over these additional parameters. Such multi-dimensional RMs quickly become unwieldy if implemented purely as a SRM. Hence hybrid SRM-PRM solutions are utilized which is beyond the present scope. For simplicity of development within this paper, only the two dimensional spatial RM will be considered with the understanding that this can be generalized to extend over an arbitrary number of dimensions. RSS influencing factors not represented in the RM such as small scale multipath are not modeled and thereby described in a statistical sense represented by random variables.

For completeness it should be added that there are recent developments in using channel state information (CSI) [29,30,31,32] instead of RSS. This has shown promise for wide bandwidth signals such as new generations of Wi-Fi, LTE and UWB with large bandwidths. Carrier phase information of CSI observables can provide additional signal features such as distinguishing between line of sight (LOS) and non-line of sight (NLOS). This can be represented as an additional dimension in the RM based on the Ricean K-factor. Detection of LOS further allows for a possible bearing angle observation between the MWD and AP [33]. However, the processing of CSI is more complex and often ill posed. Often only the magnitude of the CSI is considered with the phase ignored which is tantamount to RSS representation.

The utility of the FI in the MWD location estimation based on RSS observables is best introduced by first considering propagation along a single free space dimension. The power of the transmitted signal measured by a receiver antenna at a distance of u meters from the transmitter antenna is denoted as $P_{rx}\left(u\right)$. For convenience, this is normalized such that the power at a distance of one meter from the transmit antenna is $P_{o}$ such that $P_{o}≜P_{rx}\left(1\right)$. Assuming free space propagation with a path loss exponent of two results in the relation of

(1) $$P_{rx}\left(u\right)=P_{o}u^{-2}$$

Clearly the details of the antenna such as near field effects and polarization are ignored in this idealized expression as is the absorption effects of the person carrying the MWD and scattering off of objects in the vicinity of the propagation path. As the propagation channel is reciprocal, the RSS could equally well be measured by the stationary AP with the MWD transmitting. However, to simplify the discussion in this paper, the AP will always be assumed to transmit and the MWD receive such that the MWD generates the RSS observation.

Generally, the RSS is expressed in dBm for which it is convenient to define r as the generic RSS observable which is expressed as

(2) $$\begin{array}{l} r=10\log_{10}\left(P_{rx}\left(u\right)\right)\\ r=10\log_{10}\left(P_{o}\right)-20\log_{10}\left(u\right) \end{array}$$

In a linearized parametric model this is approximated by a first order polynomial in u as

(3) $$r\left(u\right)=c_{0}+c_{1}u$$

**Figure 1:**
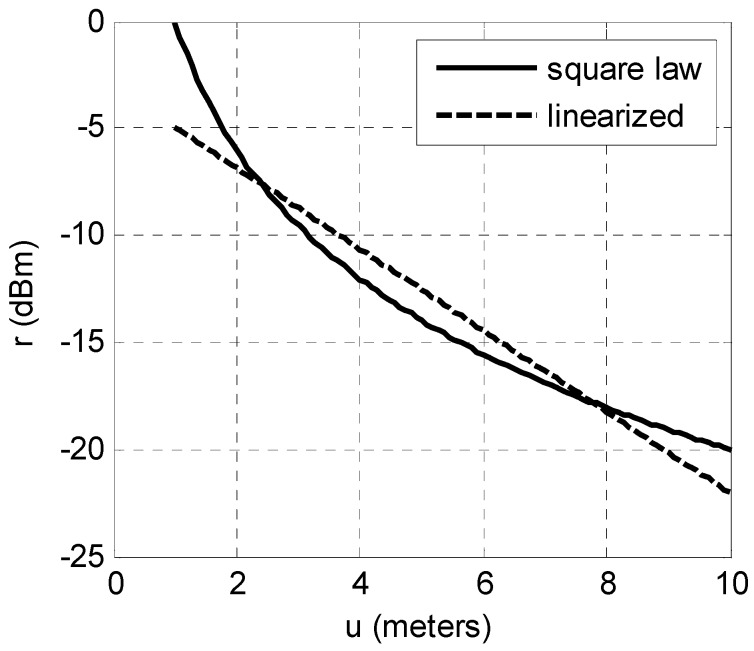
Receiver signal strength (RSS) model error due to linearization.

The linearized RSS of Equation (3) is compared with Equation (2) in Figure 1 which reveals a tolerable discrepancy. The linear form of Equation (3) is attractive as the processing is highly simplified and the PRM then reduces to only two parameters per AP. If data from pre-surveying of the RSS field is scant then the simplified two parameter model is advantageous.

The FI of the RSS observation is related to the sensitivity of the RSS to changes in the distance parameter u and the uncertainty or noise of the observation. To apply the FI, the probability density function PDF of the RSS observable conditioned on the parameter is required which is denoted as p(r|u). The FI is then computed as

(4) $$J\left(u\right)=E_{r}\left[{\left(\frac{\partial }{\partial u}\ln\left(p\left(r|u\right)\right)\right)}^{2}\right]$$

where $E_{r}\left[\cdot \right]$ denotes the expected value with respect to the random variable r. Note that ${\left(\frac{\partial }{\partial u}\ln\left(p\left(r|u\right)\right)\right)}^{2}$ increases the more sensitive p(r|u) is to changes in the parameter u resulting in higher FI. The significance of the FI is that the inverse, $J{\left(u\right)}^{-1}$, is the CRB of the minimum variance estimator of the parameter u within the family of unbiased estimators. The significance in the present context is that the lower bound of the MWD location estimation is directly computable from the FI without having to specify anything regarding the location estimation algorithm itself. This bound is simply a function of the set of observables utilized by the estimator. A caveat is that the estimator has to be unbiased which is generally too restrictive in terms of practically implemented algorithms such as MLE based algorithms. Fortunately, it can be shown that MLE based algorithms are asymptotically unbiased and asymptotically minimum variance such that the CRB applies [4].

To proceed, an assumption regarding the RSS conditional PDF is required. Typically the conditional uncertainty of the RSS observation is represented by a normal random variable such that

(5) $$r∼N\left(r|r_{o}(u),{\sigma_{r}}^{2}\right)$$

where the notation ~ implies the PDF of the left hand variable and N(r|a,b) denotes a normal PDF of the random variable r conditioned on the parameters (a,b) wherein a denotes the mean and b the variance. Hence in Equation (5), $r_{o}\left(u\right)$ describes the mean of the RSS observation and is a function of the conditioning variable u and the deviation of the RSS observation is $\sigma_{r}$. That is, $r_{o}\left(u\right)$ is the deterministic component of the RSS model and $\sigma_{r}$ is the deviation of the uncertainty or non-modelled components. The normal PDF shape is generally justified on three accounts. The first is that there are typically several independent components of the uncertainty and several independent RSS observations that are combined for the location estimation. The second is that the relevant evaluations are within the neighborhood of the central kernel of the normal PDF as opposed to the tails. The third is that the subsequent MLE processing of the location estimation implies further combining and therefore the outcome is less sensitive to the actual shape of p(r|u).

Assuming the validity of the normal PDF in Equation (5) and that $r_{o}(u)$ is an accurate model representation then the FI given in Equation (4) with θ=u evaluates to

(6) $$J_{r}\left(u\right)=\frac{1}{\sigma_{r}^{2}}{\left|\frac{dr_{o}\left(u\right)}{du}\right|}^{2}=\frac{1}{\sigma_{r}^{2}}{\left(\frac{20}{u\ln\left(10\right)}\right)}^{2}.$$

The deviation of the estimate of u will be denoted as $\sigma_{u}$ is expressed as

(7) $$\sigma_{u}>\frac{1}{\sqrt{J_{r}}}=\frac{\sigma_{r}\ln\left(10\right)}{20}u$$

which is simply proportional to the radial distance u. Equation (7) is expressed as a lower bound but as stated, if biased estimators are admitted, then this inequality is not strictly valid.

Equation (6) is of significant interest in that it states that the FI provided by the RSS observation is simply proportional to the square of the gradient of the deterministic component model, $r_{o}\left(u\right)$ divided by the variance of the uncertainty of the RSS observation. The utility of the FI of practical significance is that the inverse is a reasonable approximation to the lower bound of the deviation of the estimate of the MWD location, which in this simple case is proportional to u. Furthermore it is noted that in a practical scheme with multiple APs and hence multiple RSS observations that such samples are weighted and combined to form the estimate of the MWD location. The weight is typically heuristically selected as being proportional to the power level of the signal itself which is given directly from the RSS. From Equation (7) it is observed that $J_{r}$ varies as $u^{-2}$ which for the simple model with a path loss exponent of 2 implies that $J_{r}$ varies linearly with RSS. Consequently weighting based on FI is equivalent to weighing based on RSS.

**Figure 2:**
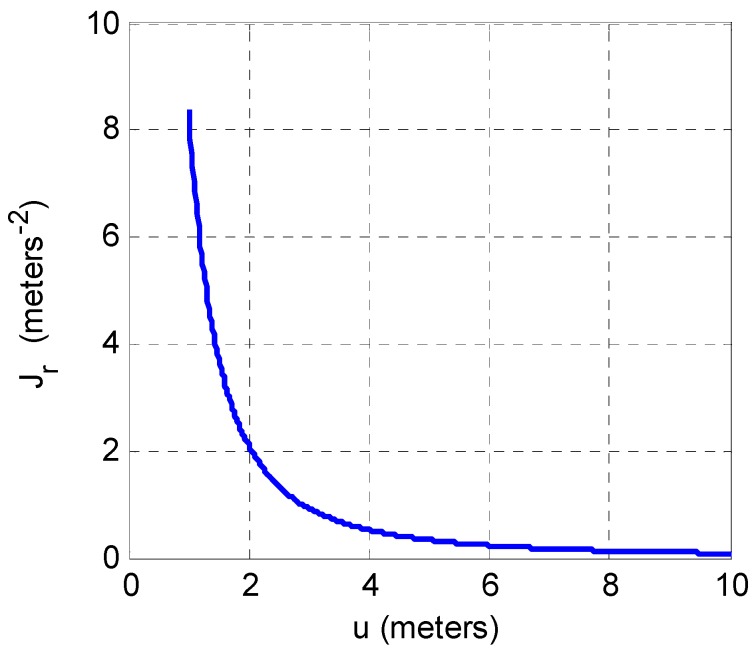
Fisher Information of a single RSS observation with $\sigma_{r}=3\;\mathrm{dB}$.

Furthermore, considering the plot of $J_{r}$ as a function of u as given in Figure 2 it is observed that FI decreases rapidly towards zero as u increases. Practical application of this observation is that the significance of an RSS observation in idealized free space propagation conditions dwindles quickly as the range between the AP and MWD increases. However, for smaller values of u close to the AP, it is reasonable to approximate the signal propagation as in free space with a loss exponent of 2. Putting this together, in the vicinity of the AP where free space propagation is a valid model, the FI weighting is equivalent to the RSS weighting and this is also the region in which the FI is the largest. Consequently, the rather heuristic weighing of RSS samples proportional to signal power level is justified in the context of the FI.

**Figure 3:**
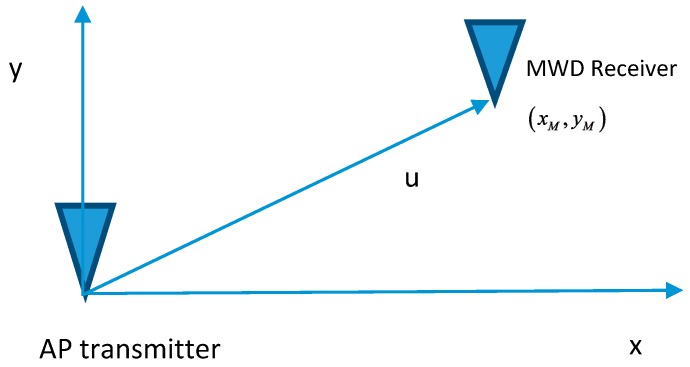
Two dimensional wireless link.

Next consider the AP transmitting into a two dimensional space as illustrated in Figure 3. Propagation is again based on the idealized free space propagation model. The MWD can move within this two dimensional space with a radial distance of u between the MWD and the AP and a location at $\left(x_{M},y_{M}\right)$. Clearly $\left(x_{M},y_{M}\right)$ cannot be jointly estimated from a single RSS measurement by the MWD. However, the Fisher Information Matrix (FIM) can be computed for a single RSS observation with the FI components evaluated for each displacement variable.

The FIM for the single RSS observation is denoted as $J_{r}$ which is expressed as

(8) $$J_{r}=\left[\begin{array}{cc} J_{xx} & J_{xy}\\ J_{yx} & J_{yy} \end{array}\right]=\left[\begin{array}{cc} {g_{x}}^{2} & g_{x}g_{y}\\ g_{x}g_{y} & {g_{y}}^{2} \end{array}\right]$$

where

(9) $$\begin{array}{l} g_{x}=\left|\frac{dr\left(u\right)}{du}\frac{du}{dx}\right|=\left|\frac{dr\left(u\right)}{du}\right|\frac{x}{u}\\ g_{y}=\left|\frac{dr\left(u\right)}{du}\frac{du}{dy}\right|=\left|\frac{dr\left(u\right)}{du}\right|\frac{y}{u} \end{array}$$

**Figure 4:**
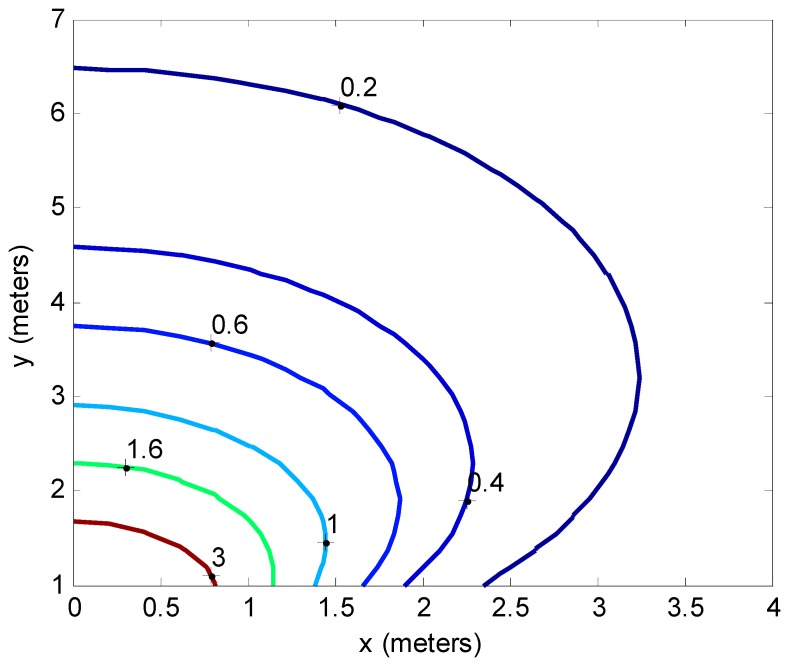
Plot of $J_{yy}$ for two dimensional location estimation (units of label numbers are m^−2^).

Consequently

(10) $$\begin{array}{l} J_{xx}=\frac{1}{\sigma_{r}^{2}}{\left(\frac{20}{\ln\left(10\right)}\right)}^{2}\frac{x^{2}}{u^{4}}\\ J_{yy}=\frac{1}{\sigma_{r}^{2}}{\left(\frac{20}{\ln\left(10\right)}\right)}^{2}\frac{y^{2}}{u^{4}} \end{array}$$

which decays quickly with increased radial distance from the AP. A contour plot of $J_{yy}$ is given in Figure 4. n passing, note that the FI is zero in the direction perpendicular to the radial vector between the MWD and AP as there is no signal variation. In Equation (8) note that $\left|J_{r}\right|=0$ regardless of the values of $g_{x}$ and $g_{y}$, indicating that the FIM is singular and cannot be inverted. This implies that the parameters $\left(x_{M},y_{M}\right)$ cannot be jointly estimated from the single RSS observation as expected.

To get a joint estimate of $\left(x_{M},y_{M}\right)$ at least two RSS observations are required from spatially separated APs. The FIM associated with each AP can be combined such that the aggregate FIM of the combined observations becomes non-singular. To develop this, denote the array of N RSS observations corresponding to N APs distributed within the two dimensional space as $r=\left\{\begin{array}{cccc} r_{1} & r_{2} & \cdots & r_{N} \end{array}\right\}$ which is conditioned on the MWD location parameters denoted as $\theta =\left(x_{M},y_{M}\right)$. It is further assumed that the uncertainties associated with each of the RSS measurements are mutually independent as is generally justified which allows the factorization of $p\left(r|\theta \right)=\prod_{n=1}^{N}p\left(r_{n}|\theta \right)$. This is the naïve Bayesian assumption of conditional independence which needs to be verified for each new scenario. If for example, the APs are not physically separated and use the same wireless carrier then the conditional independence assumption is invalid. Denote the component FIM for the *n*th AP as $J_{n}$ and $J_{T}$ as the aggregate FIM representing the combined set of N RSS observables. The parameter vector in this case is $\theta =\left(x_{M},y_{M}\right)$ wherein, for convenience, denote $\theta_{i}$ as the *i*th element of θ and ${\left[J_{T}\left(\theta \right)\right]}_{i,j}$ denotes the (*i*,*j*) element of $J_{T}$. Based on the FIM formulation

(11) $${\left[J_{T}\left(\theta \right)\right]}_{i,j}=-E_{r}\left[\left(\frac{\partial^{2}}{\partial \theta_{i}\partial \theta_{j}}\ln\left(\prod_{n=1}^{N}p\left(r_{n}|\theta \right)\right)\right)\right]$$

which is expanded as

(12) $$\begin{array}{l} {\left[J_{T}\left(\theta \right)\right]}_{i,j}=-E_{r}\left[\sum_{n=1}^{N}\frac{\partial^{2}}{\partial \theta_{i}\partial \theta_{j}}\ln p\left(r_{n}|\theta \right)\right]\\ =-\sum_{n=1}^{N}E_{r}\left[\frac{\partial^{2}}{\partial \theta_{i}\partial \theta_{j}}\ln p\left(r_{n}|\theta \right)\right]=\sum_{n=1}^{N}{\left[J_{n}\left(\theta \right)\right]}_{i,j} \end{array}$$

with the conclusion that

(13) $$J_{T}\left(\theta \right)=\sum_{n=1}^{N}J_{n}\left(\theta \right).$$

Equation (13) has practical significance in the context of evaluating the impact of individual RSS observations. That is, the contribution of an individual AP can be assessed in terms of the aggregate information for each location parameter. This implies that trade-offs associated with placement of the set of N APs can be quantified by calculating the component FIM of $J_{n}$.

For notational convenience, denote the components of $J_{T}$ as $J_{T}=\left[\begin{array}{cc} J_{xx} & J_{xy}\\ J_{yx} & J_{yy} \end{array}\right]$. The inverse of $J_{T}$ is the covariance matrix of the estimates of the variables $\left(\hat{x_{M}},\hat{y_{M}}\right)$, denoted as $Q_{\theta }$ and given as

(14) $$Q_{\theta }={J_{T}}^{-1}={D_{p}}^{2}\left[\begin{array}{cc} 1/J_{xx} & -J_{xy}\\ -J_{yx} & 1/J_{yy} \end{array}\right]$$

where $D_{p}$ is the so called *dilution of precision factor*, given as

(15) $$D_{p}={\left(1-\frac{{J_{xy}}^{2}}{J_{xx}J_{yy}}\right)}^{-1/2}.$$

The CRB of the variance for the estimation of the parameters $x_{M}$ and $y_{M}$, denoted as $\sigma_{xM}^{2}$ and $\sigma_{yM}^{2}$ respectively are given by the diagonal components of $Q_{\theta }$ as

(16) $$\begin{array}{l} \sigma_{xM}^{2}={D_{p}}^{2}/J_{xx}\\ \sigma_{yM}^{2}={D_{p}}^{2}/J_{yy} \end{array}$$

The $\sqrt{CRB}$ of the magnitude of the location error is then evaluated as

(17) $$\sqrt{\sigma_{xM}^{2}+\sigma_{yM}^{2}}=D_{p}\sqrt{\frac{1}{J_{xx}}+\frac{1}{J_{yy}}}$$

## 3. FI Analysis of MWD Location Estimation

**Figure 5:**
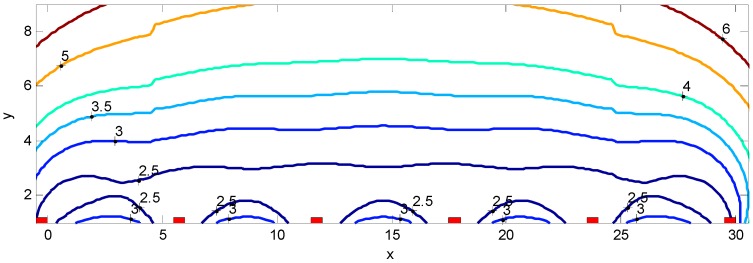
Deviation of the location estimate of the mobile wireless device (MWD) based on six access points (Aps) shown as red rectangles separated by 6 m and the contour labels indicating the MWD location deviation in meters.

In this section the FI preliminaries outlined in the previous section will be applied in the performance assessment of the two dimensional MWD location estimation. Initially an example based on N = 6 APs along the x axis separated by a distance d = 6 m with the MWD located at $\left(x_{M},y_{M}\right)$ is evaluated. Assume that the six RSS observations have a deterministic component as given by Equation (2) with a deviation of $\sigma_{r}=4\;\mathrm{dB}$. Further assume that the RSS measurements are conditionally independent. The component FIMs can then be evaluated and totaled as giving the aggregate FIM $J_{T}$ with the CRB of the location estimate corresponding to the diagonal components of ${J_{T}}^{-1}$. Figure 5 shows the deviation of the location estimate as a contour plot with the contour labels indicating the location estimate deviation in meters. The red patches show the location of the APs. The deviations increase significantly as the MWD moves away from the row of APs.

In Section 4, experimental results for a commercial office area will be provided which includes measurements of the components of $\sigma_{r}$ justifying the choice of $\sigma_{r}=4\;\mathrm{dB}$ for the results given in Figure 5 which are calculated based on Equation (17). As observed, the location deviation increases significantly as the MWD is moved away from the row of APs. This indicates that reasonable (say less than 3 m deviation) point location accuracy is possible only when the MWD is within several meters of the row of APs.

**Figure 6:**
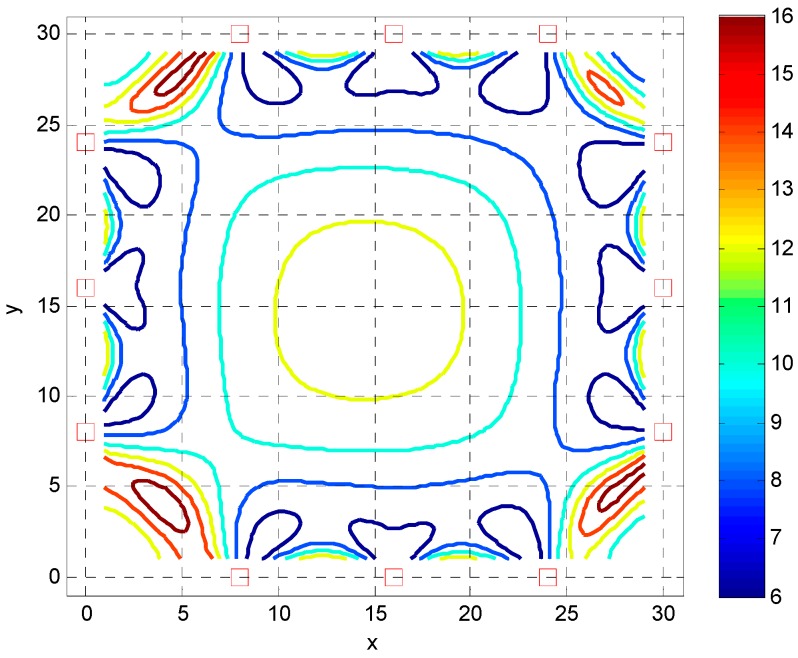
Cramer Rao Bound (CRB) of the magnitude of location estimate as a function of $J_{xx}\left(x_{MWD},y_{MWD}\right)$ with red squares indicating the location of the APs with 8 m separation.

**Figure 7:**
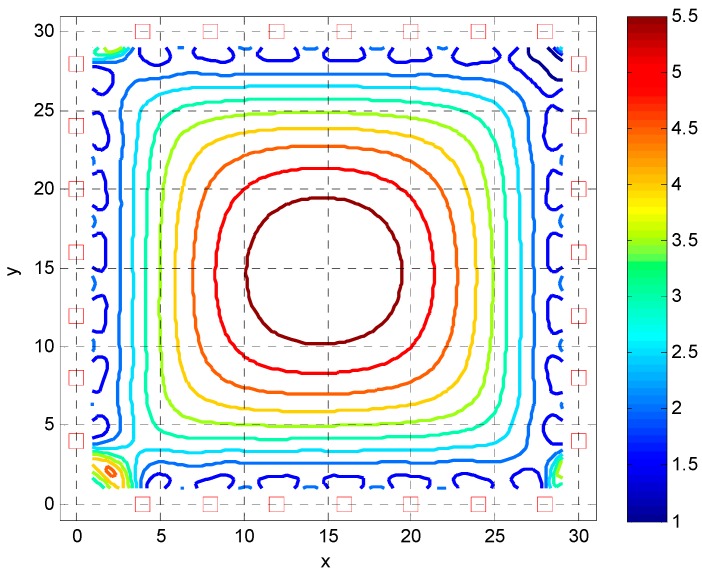
CRB of the magnitude of location estimate as a function of $J_{xx}\left(x_{MWD},y_{MWD}\right)$ with red squares indicating the location of the APs with 4 m separation.

As an additional example, consider the FI analysis of the previous example is expanded to a two dimensional square room with APs evenly spaced about the perimeter. The dimension of the room is 30 m with a string of APs along the wall perimeter with a spacing of 8 m. The deviation of the RSS measurements is $\sigma_{r}=4\;\mathrm{dB}$ as before. Figure 6 shows the location of the APs and the CRB of the magnitude of the location error. As observed, the deviation of the location estimate becomes large away from the wall and reaches a maximum in the center of the room. The performance is only somewhat satisfactory close to the perimeter of the wall even with a moderate number of APs contributing to the RSS observables. Also note that in the corners of the room, the CRB becomes large due to the large dilution of precision term. For an acceptable location deviation a high spacing of APs of 4 m separation is required. Results of this are given in Figure 7 showing similar characteristics as in Figure 6 but with a more usable location deviation.

As demonstrated by examples in this section, the FI is of practical utility in quantifying the information content of a set of RSS samples resulting in the CRB of the MWD position estimation. Although the modeling is highly simplistic, the calculated CRB as a function of position, is commensurate with practical experience of RSS based location estimation performance in typical building environments.

## 4. Experimental Verification of FI Analysis

The practical utility of FI analysis in the context of MWD location estimation is considered in this section which is based on an extensive set of RSS measurements taken in a mid-size office area of 15 by 30 m. There are three objectives: (1) Explore the nondeterministic components of the RSS measurements and justify a value of $\sigma_{r}$; (2) Provide a practical method of calculating the FI based on a sampled RM; (3) Show that the FI analysis results in a CRB of the location estimate deviation that is commensurate with the actual deviation that is determined based on using a MLE estimator operating the vector of RSS samples. Before embarking on these objectives, the experimental measurement facility will be described.

### 4.1. Measurement of RSS Field

**Figure 8:**
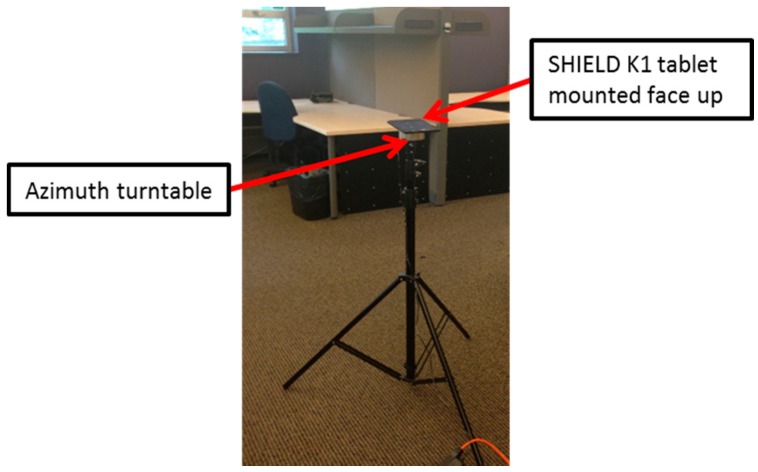
SHIELD K1 tablet mounted on tripod for RSS data collection.

**Figure 9:**
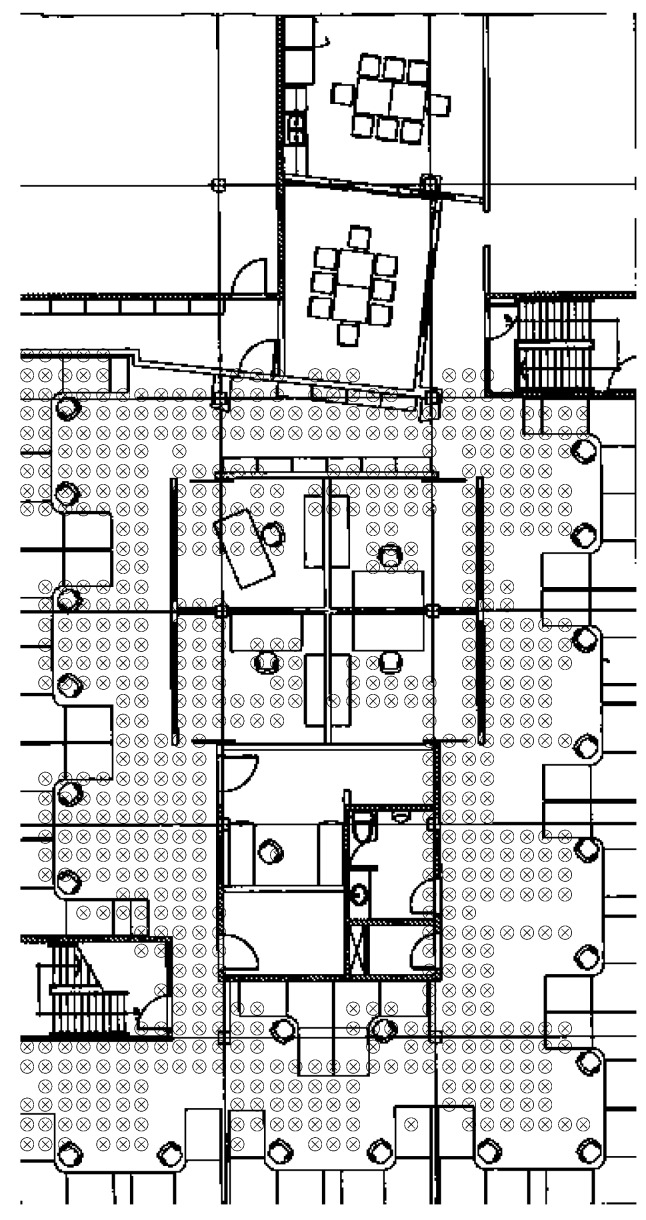
Office area with RSS sample points indicated by the markers.

A set of 20 APs distributed about the office area each mounted at a height of 2 m transmit Wi-Fi signals in the 2.4 GHz and 5 GHz bands. The MWD used for this data collection is a NVIDIA SHIELD K1 tablet with a built in RSS scanner that provides RSS measurements for each MWD-AP link at a rate of approximately 1 Hz. Figure 8 shows a photo of the MWD tablet mounted on a small azimuth turntable which is in turn mounted on a tripod. Figure 9 shows the floor map of the office area with all of the RSS sampling positions indicated by the small circles with x’s. In all, there are 714 sampling positions residing on a uniform grid with spacing of 50 cm. At each location an average of 30 consecutive RSS samples for each Wi-Fi signal was stored which will be referred to herein as an RSS sample subset. In total, over a million valid RSS samples were recorded. Two sets of data were collected with the first based on the tablet being stationary and the second with the tablet rotated slowly in azimuth.

### 4.2. Measurement of Non-Modeled RSS Sample Factors

**Figure 10:**
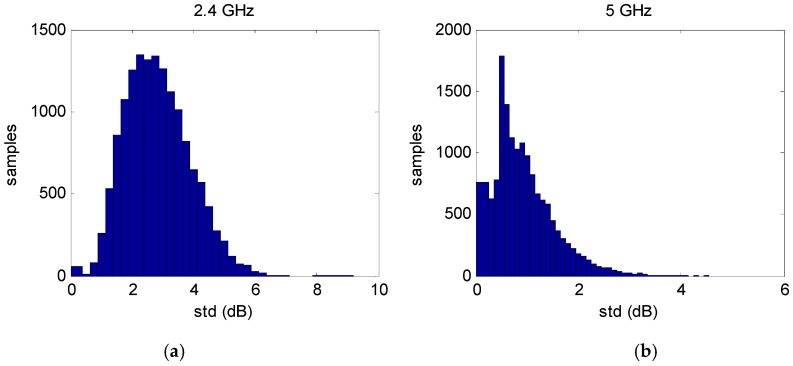
Deviation of the RSS within the subsets (**a**) 2.4 GHz Wi-Fi data; (**b**) 5 GHz Wi-Fi data.

For each sample subset consisting of approximately 30 RSS samples, the mean and deviation were calculated. Care was taken during the measurements to ensure that the propagation environment was static such that only the nominal radio interference was a factor. It was found that the deviation of the RSS within a sample subset was a mild function of the RSS value for the 5 GHz Wi-Fi. That is, the deviation decreased slightly as the average RSS increased. However, this was not significant and subsequently ignored. For the 2.4 GHz band there was no dependence of the subset deviation with average RSS. Figure 10 shows the histogram of the RSS deviations within the sample sets for 2.4 GHz and 5 GHz. The average temporal deviation of 2.4 GHz Wi-Fi is therefore about 3 dB where for 5 GHz it is about 1 dB.

**Figure 11:**
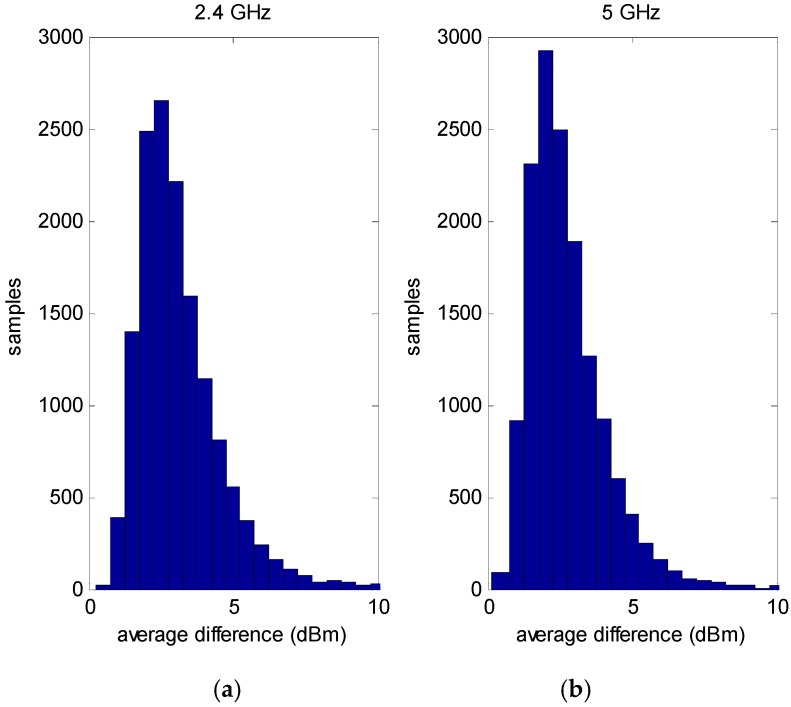
Average absolute difference of the RSS of a sampling point relative to neighbors within a 0.8 m radius. (**a**) 2.4 GHz Wi-Fi data; (**b**) 5 GHz Wi-Fi data.

Next consider the uncertainty resulting from the non-modeled small scale multipath. To determine this, the non-rotated data set was used again. This time the change in the mean RSS of all of the RSS subsets relative to the closest neighbor sampling points was calculated. In other words, at each sampling point in Figure 9, neighboring sampling points within a 0.8 m radius circle were considered. As the grid spacing of the sampling points is 0.5 m, typically eight adjacent sampling points were included in calculating this average difference. Note that as the mean of the individual RSS subsets was used, the temporal variation was averaged out. Figure 11 shows the histogram of the magnitude of the difference in RSS due to the spatial variation of the sampling point. Note that the histograms for the 2.4 GHz and the 5 GHz data are very similar. This should be expected as the temporal variations are averaged out as the mean of the sub sample set is used. Also the small scale multipath between the grid of sampling points of 50 cm is several wavelengths for either 2.4 or 5 GHz and therefore is generally statistically independent. The average difference of the RSS means for the sampling points within a 0.8 meter radius was 3 dB for 2.4 GHz and 2.7 dB for 5 GHz. Hence we can ascribe an average deviation of about 3 dB due to the non-modeled small scale multipath effect.

**Figure 12:**
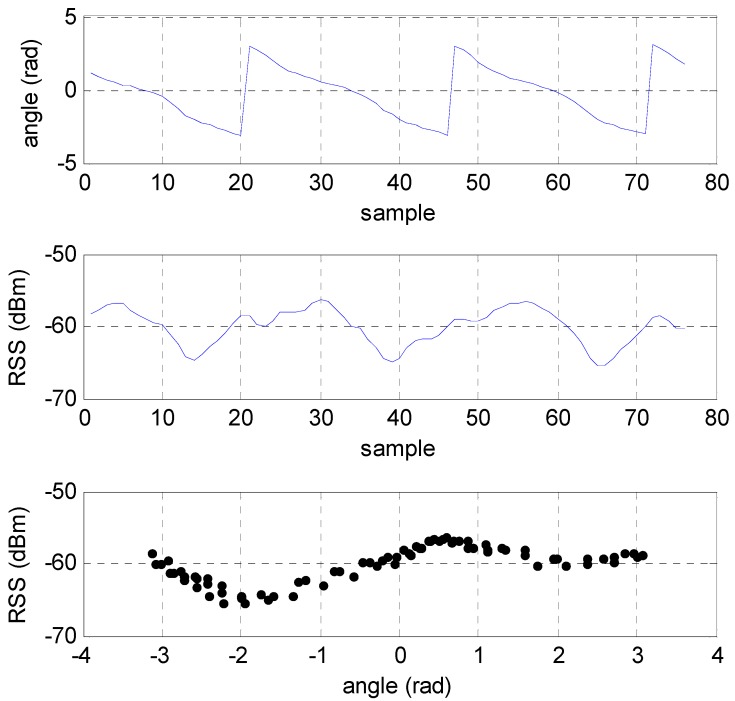
Variation of RSS due to antenna orientation. Top plot is the azimuth angle approximated by the tablet magnetometer and accelerometer output. Middle plot is a segment of the RSS samples collected for the 2.4 GHz AP channel and the bottom plot is the RSS as a function of the azimuth angle.

Next the uncertainty due to the non-modeled antenna orientation was considered by using the rotated data. This antenna orientation variation depended on the ratio of the LOS and NLOS components of the AP signal received at the MWD. An example of where this ratio was higher at sampling points several meters from the AP is given in Figure 12. Note that the turntable azimuth angle was estimated by the heading output of the tablet which is not highly accurate but sufficient to approximate the RSS deviation due to azimuth orientation. In this example the deviation was on the order of 7 dB. Higher deviations were observed for sampling points where the tablet was placed closer to the AP such that the propagation was more LOS. Likewise, if the tablet was further than about 5 m from the AP, then the variation in RSS due to azimuth was less as propagation was more NLOS and dominated by multipath components. Typical root mean square deviation was on the order of 3 dB.

**Figure 13:**
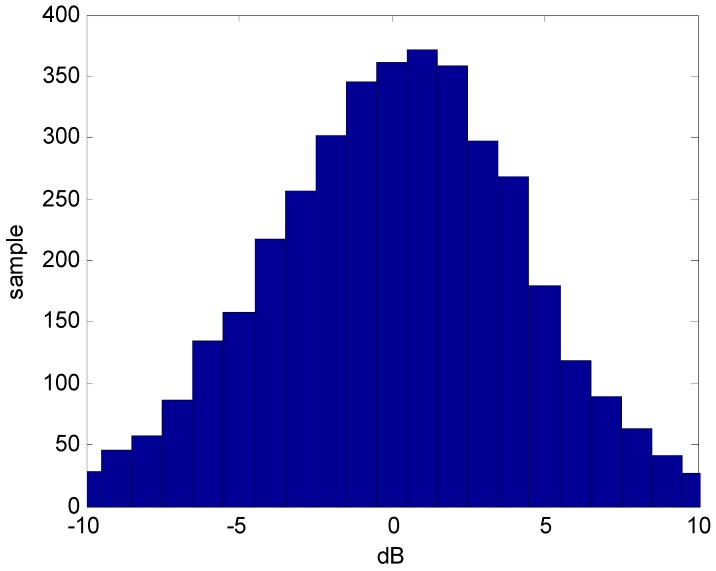
Histogram of the variation of RSS for combined temporal, antenna orientation and body position.

Finally, the deviation due to body absorption combined with azimuth rotation and position of the tablet were evaluated experimentally. This was done by collecting RSS samples while the tablet was moved alternately between an out front position with the tablet screen roughly face up towards the ceiling and an upright position held to the chest roughly representative of a smart phone in a shirt pocket. While alternating between these two positions, the person slowly rotated in azimuth collecting about 10 m of samples, equivalent to approximately 800 RSS samples per Wi-Fi channel. While the position of the tablet was easily determined from the attitude and heading indicators of the tablet, there was no meaningful correlation with the RSS values. Therefore, the RSS deviations within each Wi-Fi channel sample sub-group were combined into an overall histogram as shown in Figure 13. This indicates that there was roughly a 5 dB RSS deviation that should be attributed to the combined uncertainty of the orientation, absorption and temporal variation.

The deviations are summarized in the Table 1.

### 4.3. Calculation of FI from Sampled RSS Values

In this section the interpolation method used to construct the SRM and PRM is given. The FI is calculated from the SRM with assumptions regarding the RSS sample deviation based on the statistics of non-modeled components outlined in the previous subsection. The SRM is generated from the sampled data by a Gaussian kernel integration interpolation method as follows. Assume a uniformly spaced rectangular grid of points with indices of *i* and *j* and coordinate tuples of $\left(x_{g,i,j},y_{g,i,j}\right)$ that cover the office floor area of Figure 9. Then let *k* be the index of the survey points with coordinates of $\left(x_{s,k},y_{s,k}\right)$. Denote $R_{s,k,m,n}$ as the *m*th RSS sample data at $\left(x_{s,k},y_{s,k}\right)$ corresponding to the *n*th AP. In the current case *k* = 1,…,714 corresponding to the array of sampled points in Figure 9, *m* = 1,…,30 and *n* = 1,…,20. The Gaussian interpolation weighting kernel, $w_{i,j,k}$ is given as

(18) $$w_{i,j,k}=\exp\left(-\alpha \left({\left(x_{g,i,j}-x_{s,k}\right)}^{2}+{\left(y_{g,i,j}-y_{s,k}\right)}^{2}\right)\right).$$

Here α is the interpolation parameter of the kernel. The value of α depends on how much spatial filtering is desired. When α is smaller, more smoothing is provided by the kernel. Selecting α of about 0.5 is a reasonable compromise as the deviation of the interpolation kernel is then one meter. This maintains the low spatial frequency variations of the path loss and the shadowing but removes most of the variation due to the small scale multipath. Let $W_{i,j}$ be the accumulated weight and $R_{i,j,n}$ be the accumulated weighted RSS for the *n*th AP at the grid point $\left(x_{g,i,j},y_{g,i,j}\right)$ which results in the SRM. Then the interpolation algorithm sums over all of the sample points as

(19) $$\begin{array}{l} R_{i,j,n}=\frac{1}{MW_{i,j}}\sum_{m=1}^{M}\sum_{k}w_{i,j,k}R_{s,k,m,n}\\ W_{i,j}=\sum_{k}w_{i,j,k} \end{array}.$$

The PRM is derived directly from the SRM based on a linear parametric model with parameters of $\left\{x_{AP},y_{AP},c_{0},c_{1}\right\}$ given as

(20) $$\begin{array}{l} u_{k}=\sqrt{{\left(x_{s,k}-x_{AP}\right)}^{2}+{\left(y_{s,k}-y_{AP}\right)}^{2}+\Delta h^{2}}\\ r_{k}=c_{0}+c_{1}u_{k} \end{array}$$

where Δh is the difference in height of the ceiling mounted AP and the height of the MWD. The best fit parameters were determined using a constrained optimizer (Matlab lsqnonlin).

Finally the FI for the *n*th AP is determined over the rectangular grid based on the same procedure as the derivation of the SRM. Recall the FIM in Equation (8) but now define this for the *n*th MWD-AP link at the sample point $\left(x_{g,i,j},y_{g,i,j}\right)$ as

(21) $$J_{i,j,n}=\left[\begin{array}{cc} J_{xx,i,j,n} & J_{xy,i,j,n}\\ J_{yx,i,j,n} & J_{yy,i,j,n} \end{array}\right]=\frac{1}{\sigma_{r,n}^{2}}\left[\begin{array}{cc} {g_{x,i,j,n}}^{2} & g_{x,i,j,n}g_{y,i,j,n}\\ g_{x,i,j,n}g_{y,i,j,n} & {g_{y,i,j,n}}^{2} \end{array}\right].$$

Here $\sigma_{r,n}^{2}$ is the variance associated with the SRM components of $R_{i,j,n}$. $g_{x,i,j,n}$ and $g_{y,i,j,n}$ are the gradient components of the RM which can be determined directly from the SRM at the sample point. In the current implementation a better numerical method used is to interpolate the surveyed RSS data directly as

(22) $$\begin{array}{l} g_{x,i,j,n}=\frac{1}{MW_{i,j}}\sum_{m=1}^{M}\sum_{k}w_{x,i,j,k}R_{s,k,m,n}\\ g_{y,i,j,n}=\frac{1}{MW_{i,j}}\sum_{m=1}^{M}\sum_{k}w_{y,i,j,k}R_{s,k,m,n} \end{array}$$

where

(23) $$\begin{array}{l} w_{x,i,j,k}=2\alpha \left(x_{g,i,j}-x_{s,k}\right)w_{i,j,k}\\ w_{x,i,j,k}=2\alpha \left(y_{g,i,j}-y_{s,k}\right)w_{i,j,k} \end{array}$$

are the gradient components of the Gaussian weighting kernel of $w_{i,j,k}$. With this method, the FI for a specific AP can be computed directly from the arbitrary set of sampled RSS points. The total FI is then merely the sum of the N component FIMs.

### 4.4. Comparison of CRB Based on FI and MLE Deviation

In this section an example of the SRM and PRM for a specific AP will be given. Then the total FIM is calculated from all of the APs and from this the CRB of the deviation of the MWD location estimation. This is compared to the deviation of the MWD location estimation calculated directly from the RSS samples of the APs using a weighted MLE approach. For the MLE deviation the tablet was positioned at each of the sample points in Figure 9 with the Wi-Fi scanner collecting the vector of N RSS samples for each 2.4 GHz AP with position parameters evaluated from the lowest MLE error. The MLE weighting was proportional to the signal power level of the APs with zero weight assigned to APs with RSS of less than −70 dBm.

**Figure 14:**
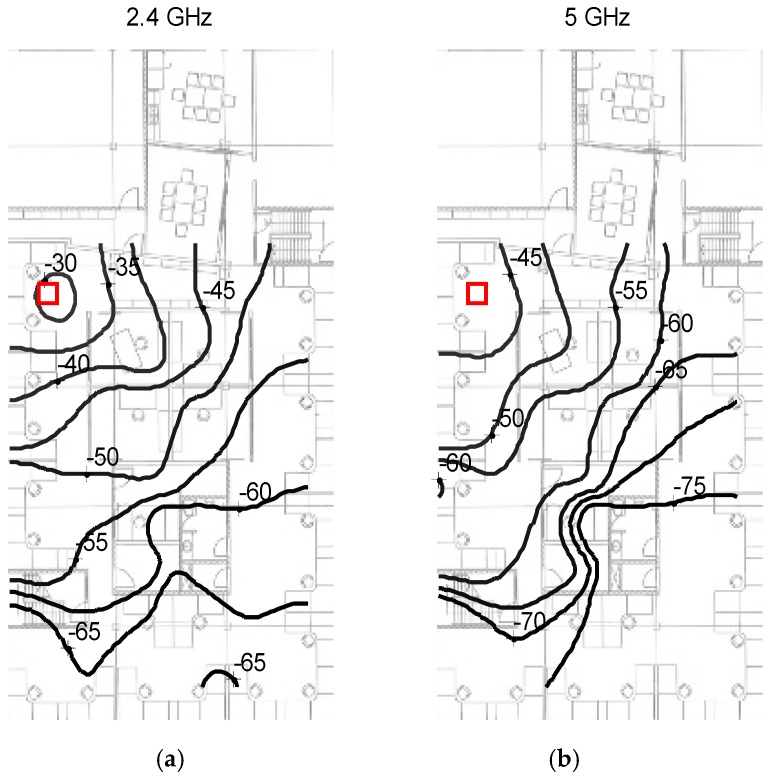
Office radio map (RM) for 2.4 and 5 GHz for Wi-Fi channel, red square is location of AP, numbers are given in dBm (**a**) 2.4 GHz Wi-Fi data; (**b**) 5 GHz Wi-Fi data.

Figure 14 gives an example of the interpolated SRM for the 2.4 GHz and 5 GHz Wi-Fi channels. As observed, the constant RSS contours close to the AP are roughly circular indicating approximate LOS propagation. Further away from the AP the contours become distorted indicative of large scale multipath and shadowing. Also note that the RMs of the 2.4 and 5 GHz have similar behavior (within an offset constant) near the AP but behave rather differently further away from the AP which is distorted by shadowing, excess penetration and diffraction propagation effects.

**Figure 15:**
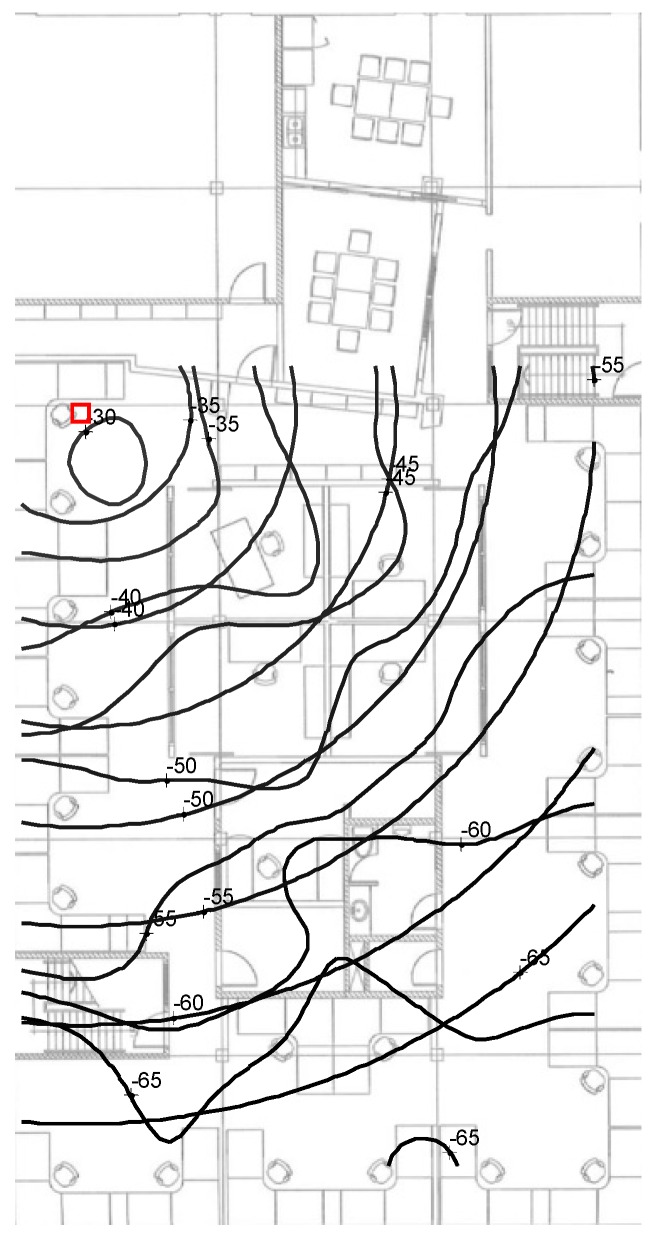
Superposition of the parametric RSS model (concentric circles) and the non-parametric RM with red square the estimated position of the AP at 2.4 GHz.

The linear parametric model of the PRM is compared to the SRM in Figure 15 with the uniform concentric circles corresponding to the PRM. As observed, the fit is reasonable near the AP as indicated by the red square but deteriorates for larger ranges. The discrepancy between the PRM and SRM near the AP is within 2 dB, indicating that the LOS propagation assumption of the PRM is reasonable. Further away from the AP the discrepancy of the SRM and PRM increases as the propagation becomes more NLOS and hence not well represented by the SRM. However, the consequences of the inadequate modeling representation by the SRM in regards to the location estimation are minimal due to the MLE weighting. That is signals with low RSS or FI as in the NLOS region in this example have minimal weight.

**Figure 16:**
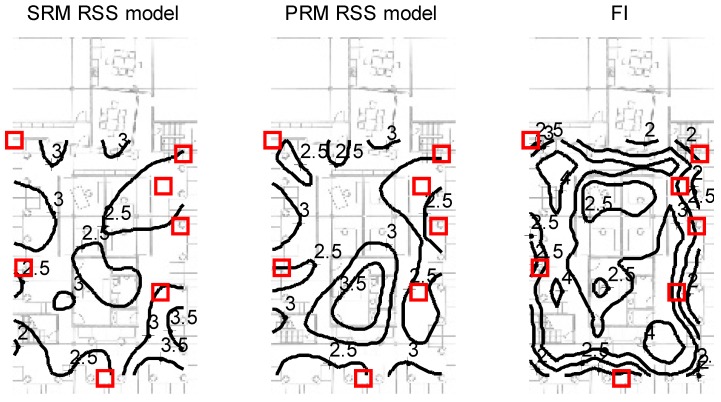
Evaluation of the MWD location error for a selection of 2.4 GHz APs indicated by the red squares. The **left** and **middle** plots are the estimation deviation based on the sampled radio map (SRM) and parametric radio map (PRM) respectively while the **right** side plot is the estimation deviation as calculated based on the Fisher Information Matrix (FIM).

As stated, the CRB of the MWD location estimate, based on the total FIM of a selection of seven APs was calculated with the results given in Figure 16 which shows contour plots of the deviation of the estimate of the MWD location with the selection of 2.4 GHz APs indicated by the red squares. The tablet was held in front of the person but the azimuth orientation was random. The left plot is the MWD location estimation deviation based on the weighted MLE with the set of SRMs corresponding to the APs indicated. Due to the uniform distribution of the selected APs and the relatively small office space, the deviation of the location estimate is fairly uniform throughout the entire office area. The middle plot is the estimation error based on the linearized PRM. Note that there is an additional uncertainty as the RM is better represented by the SRM than the PRM. This additional uncertainty results in a slight increase of the location estimation deviation. However, it is remarkable that this additional uncertainty is not all that significant. Hence for small to mid-sized building areas, a linearized parametric model is typically sufficient. However, this is not true for multi-story open atrium building areas where in such areas the features of the RSS map are inadequately represented by the linearized PRM. The right side plot of Figure 16 is the estimation deviation calculated based on the FI as evaluated using the method given above. The variations due to the non-modeled temporal, small scale multipath and azimuthal orientation uncertainty components were combined into an assumed 5 dB deviation for each RSS sample based on the measurements given in the previous subsection. Note that the contour plot of the FI deviation estimate is very similar to that of the deviations of the weighted MLE used with the SRMs and PRMs with notable incidental differences. As observed, the FI based deviation in the right hand plot is slightly higher than the average experimental deviations of the left hand plot. A plausible explanation is that the weighted MLE is not an unbiased estimator while the CRB is lower bound only for the family of unbiased estimators. Therefore while the CRB in this case is a reasonable indication of the location estimation deviation performance it is not strictly a lower bound as discussed earlier.

## 5. Conclusions

The main objective of this paper was to develop the utility of using FI as a practical tool for evaluating the potential performance of an RSS based location scheme. As highlighted, the FI is easily and robustly evaluated from a sampled RSS field. This enables the FI contribution of each wireless signal associated with an RSS observable in the context of the overall MWD location estimation scheme to be quantified. From the calculated total FI of the complete set of observations it can readily be determined if there is sufficient information to achieve a given location estimation performance and coverage. Thus as discussed, the FI serves as a guide for optimal combining of observables. Furthermore, in cases of practical interest, weighting proportional to the component FI is tantamount to sample weighting based on signal strength. Hence, while the FI analysis may not directly result in a novel combining algorithm, it provides interesting insight and justification for the weighted MLE which is typically employed for location estimation. In other words, as seen from the experimental observations, the FI diminishes quickly as the separation distance between the AP and the MWD increased. Hence in a building complex, APs closer to the MWD should be weighted more than the APs further away. It was also observed that when such weighting was applied, the location accuracy of RSS fingerprinting based on SRMs and PRMs was not significantly different. This was demonstrated with simulated results as well as experimentally based on a midsized office with distributed APs.

As developed in the paper, RSS signals are feature rich with significant inherent information related to MWD location. However, most of the feature content is impractical to use. It is only the feature components that can be robustly modeled and represented in the RM that are useful. This includes the large scale shadowing and path loss components. The small scale multipath fading, antenna orientation as well as body absorption generally requires the joint estimation of multiple nuisance parameters. Estimation of such parameters is generally difficult and not robust unless other sensors are applied or additional information is provided. In typical applications these become non-modeled features and hence part of the uncertainty or noise related with the measurement. Various noise components of the RSS observables were measured experimentally. Also discussed was the additional uncertainty created in moving from the SRM to the linearized PRM. In conclusion, the results from this paper demonstrate the merit of using FI analysis to provide intuition and insight into the problem of MWD location estimation based on RSS observables. This work can be used to provide a more unified approach to constructing and justifying combining algorithms, as well as assessing the fundamental performance limitations of a MWD location estimation system.

## Figures and Tables

**Table 1:** Summary of measured deviations of uncertainties related to the RSS measurement.

RSS Sample Uncertainty Factors	Deviation Value
temporal variation	3 dB for 2.4 GHz band and 1 dB for 5 GHz band
small scale multipath	3 dB for 2.4 GHz band and 2.7 dB for 5 GHz band
antenna orientation	moderate level of LOS about 3 dB, NLOS negligible
combined, body absorption, temporal variation and small scale multipath	roughly 5 dB in NLOS environment. In a LOS environment can be up to 20 dB
